# Gestational Exercise and Maternal and Child Health: Effects until Delivery and at Post-Natal Follow-up

**DOI:** 10.3390/jcm9020379

**Published:** 2020-01-31

**Authors:** María Perales, Pedro L. Valenzuela, Ruben Barakat, Yaiza Cordero, Mireia Peláez, Carmen López, Luis M. Ruilope, Alejandro Santos-Lozano, Alejandro Lucia

**Affiliations:** 1 Research Institue of Hospital 12 de Octubre (‘i+12’), 28041 Madrid, Spain; m.perales.santaella@gmail.com (M.P.); asantos@uemc.es (A.S.-L.); 2 Departamento de Ciencias de la Actividad Fisica y del Deporte. Universidad Camilo José Cela, 28692 Villanueva de la Cañada, Madrid, Spain; 3 Department of Systems Biology, University of Alcalá, 28801 Alcalá de Henares, Madrid, Spain; pedrol.valenzuela@edu.uah.es; 4 Facultad de Ciencias de la Actividad Fisica y del Deporte, Universidad Politécnica de Madrid, 28040 Madrid, Spain; barakatruben@gmail.com; 5 Universidad Complutense de Madrid, 28040 Madrid, Spain; yaiza.cordero@ucm.es; 6 Universidad Europeadel Atlántico, 39011 Santander, Spain; Mireiapelaez@gmail.com; 7 C.P.B. Tiemo Galván, Tres Cantos, 28760 Tres Cantos, Madrid, Spain; clmas@hotmail.com; 8 Hypertension Unit and Cardiorental Translational Laboratory, Research Institue of Hospital 12 de Octubre (‘i+12’), 28041 Madrid, Spain; ruilope@icloud.com; 9 i+HeALTH, Department of Health Sciences, European University Miguel de Gervantes, 47012 Valladolid, Spain; 10 Faculty of Sport Sciences, Universidad Europea de Madrid, 28670 Villaviciosa de Odón, Madrid, Spain

**Keywords:** obesity, hypertension, diabetes, pregnancy, training, physical activity

## Abstract

We studied the influence of pregnancy exercise on maternal/offspring cardiometabolic health until delivery and at follow-up. We pooled data from two randomized controlled trials from our group that were performed following the same methodology (one unpublished). We also collected follow-up data de novo from the participants of both trials and their offspring. In total, 1348 women with uncomplicated, singleton gestations were assigned to an intervention (*n* = 688, performing a supervised, moderate-intensity exercise program (three sessions/week)) or control group (*n* = 660). Maternal outcomes were excessive gestational weight gain (EGWG), gestational hypertension/diabetes and, at follow-up, return to pre-pregnancy weight within six months, hypertension, overweight/obesity, and other cardiometabolic conditions. Offspring outcomes were macrosomia and low-birthweight and, at follow-up, overweight/obesity, low-weight, and cardiometabolic conditions. Adherence to the intervention, which proved safe, was > 95%. Pregnancy exercise reduced the risk of EGWG, gestational hypertension, and diabetes (adjusted odds ratio (OR) and 95% confidence interval: 0.60 (0.46–0.79), 0.39 (0.23–0.67), and 0.48 (0.28–0.84)), and it was associated with a greater likelihood of returning to pre-pregnancy weight (2.37 (1.26–4.54)) and a lower risk of maternal cardiometabolic conditions (0.27 (0.08–0.95)) at the end of follow-up (median 6.1 years (interquartile range 1.8)). Pregnancy exercise also reduced the risk of macrosomia (0.36 (0.20–0.63)) and of childhood overweight/obesity during the first year (0.20 (0.06–0.63)). Our findings suggest that pregnancy exercise might protect maternal/offspring health.

## 1. Introduction

Excessive gestational weight gain (EGWG) is increasing globally [1], raising the long-term risk not only of maternal overweight/obesity [2], but also of offspring overweight/obesity [3,4] and cardiometabolic conditions [5,6]. Incident gestational hypertension and diabetes might also compromise the mother’s and offspring’s future health by increasing the risk for endothelial dysfunction, insulin resistance, hypertension, atherosclerosis, type 2 diabetes, and cardiac malformations [7,8,9,10,11]. Although low-impact moderate-intensity exercise during pregnancy reduces the risk of EGWG [12,13], and specific exercise guidelines are available [14,15,16], there is limited knowledge about the effects of gestational exercise on future maternal/offspring health. Importantly, the effects of pre-pregnancy activity habits during the post-natal period remain to be determined.

Considering the major health consequences of excess maternal weight for both the mother and the offspring and the potential health benefits of exercise, it is of medical relevance to assess the effectiveness of maternal exercise to prevent maternal and childhood cardiometabolic conditions after delivery [17]. Our main objective was to determine the effects of gestational exercise on maternal cardiometabolic health during pregnancy and on maternal/offspring health at post-natal follow-up. We also studied the influence of pre-pregnancy activity habits.

## 2. Experimental Section

### 2.1. Experimental Design

The present study complies with the recommendations of the Consolidated Standards of Reporting Trials (CONSORT) statement. We pooled data from two separate randomized controlled trials (RCT) performed by us: one from September 2007–January 2011 (NCT01790347) with data collected until delivery previously published [18] and the other with fully unpublished data (March 2012–September 2016, NCT03348332). The two trials were performed in two different medical centers located in the southern part of Madrid (Hospital Universitario de Fuenlabrada and Hospital Severo Ochoa) but shared the same methodology in terms of exercise intervention, computer-generated randomization assessment, and inclusion criteria (recruiting only women aged 18–45 years, free of contraindications for performing gestational exercise (e.g., poorly controlled diabetes/hypertension, heart disease, morbid obesity, extreme low-weight). and with uncomplicated singleton gestations) [15].

For the present study, we also collected follow-up data de novo from the participants in the two RCT and their offspring.

All procedures were performed in accordance with the Declaration of Helsinki, and the study was approved by the Institutional Review Board (Hospital Universitario Severo Ochoa, Madrid, Spain; reference # A704). Subjects signed an informed consent before their inclusion in the study.

### 2.2. Intervention

Participants in the intervention group of both separate trials performed the same supervised, light-to-moderate-intensity exercise program during weeks 9–38/39 (low-impact dance, stretching, and toning/resistance exercises with light loads (barbells, Therabands), as well as pelvic floor muscle training on three weekly 50- to 55-min sessions) (representative examples of the different exercises performed can be seen in Video S1, Table S1, and Figure S1, Supplementary Materials). Exercise intensity was controlled using heart rate monitors (women’s heart rate was kept below 60% of their age-predicted maximum heart rate (220 minus age in years)) and individual rating of perceived exertion (RPE) using the Borg’s 6–20 scale (with RPE values ranging from 10 to 12, corresponding to “fairly light” to “somewhat hard”, respectively) [18]. Both intervention and control groups received general nutrition and physical activity counseling from healthcare professionals and were not discouraged from exercising on their own [18].

For secondary analyses, participants were placed in the following four groups based on their previous exercise habits and group assignment in RCT during pregnancy (intervention (exercise)/control): (1) “previously active, intervention”, (2) “previously active, no intervention”, (3) “previously inactive, intervention”, or (4) “previously inactive, no intervention”. Women were considered to be “previously active” if they reported in the first prenatal visit having exercised on three or more days per week during the previous year, or to be “previously inactive” otherwise [19].

### 2.3. Safety

The following delivery outcomes (recorded from medical records) were analyzed to assess the safety of the exercise intervention [20]: low birthweight (<2500 g), gestational age, risk for preterm delivery, type of delivery (natural, instrumental, or cesarean), Apgar score (at 1 and 5 min after delivery), and labor times (dilatation, expulsion, and childbirth).

### 2.4. Study Outcomes

Participants were not blinded to group assignment, whereas the staff in charge of outcome data collection (from medical records or through telephone interviews) was blinded to group assignment. Maternal pregnancy outcomes (recorded from medical records) were as follows: EGWG (following Institute of Medicine criteria, i.e., > 18 kg for underweight women, > 16 kg for normal weight, > 11.5 kg for overweight, and > 9 kg for obese) [21], gestational hypertension (systolic (SBP)/diastolic blood pressure (DBP) > 140/90 mmHg at weeks 20–34) [22], and diabetes (as confirmed by a 100-g oral-glucose test at weeks 24–28) [22]. Post-natal maternal outcomes (recorded from telephone interviews with the mothers) were as follows: returning to pre-pregnancy weight within six months post delivery [23], and, at the end of follow-up (from June 2017 to September 2018), incidence of overweight/obesity (body mass index (BMI) ≥ 25 kg·m^−2^)), medically diagnosed chronic hypertension (defined as SBP/DBP ≥ 140/90 mmHg (as per European guidelines) [24] or 130/80 mmHg (as per United States (US) guidelines) [25]), or other cardiometabolic conditions collected from medical records.

Incidence of newborn’s low birth weight (<2500 g) or macrosomia (>4000 g) was recorded from medical records. The following children’s outcomes were recorded (from telephone interviews with the mother, who retrieved data from medical reports of the correspondent primary care pediatric center) at one year post delivery and at the end of follow-up (last available data): incidence of low-weight or overweight/obesity (using World Health Organization weight-for-length charts for age one year [26] and the age-and-sex BMI percentile-calculator for older ages up to six years following the 2000 Centers for Diseases Control and Prevention growth charts) [27], and incidence of cardiometabolic conditions other than overweight/obesity (up to 10 years of age, when available).

### 2.5. Statistical Analysis

Prior power calculation ensured a statistical power ≥ 90% and a level of significance of 0.05 assuming a bilateral alternative [18]. Data are shown as means ± SD (continuous variables) or percentages (dichotomous variables). Between-group baseline comparisons were performed with Student’s unpaired and chi-square tests (or Fisher’s exact test if > 20% of the cells in the cross-table had an expected frequency < 5). Logistic regression was used for risk comparisons among groups/subgroups and reported as crude results or adjusted. Thus, regression analyses of all the study outcomes up to delivery were adjusted for baseline variables (maternal age, pre-pregnancy BMI category (underweight < 18.5 kg·m^−2^, normal weight 18.5–24.9 g·m^−2^, overweight 25–29.9 kg·m^−2^, obese (>30 kg·m^−2^)), gestational hypertension during the first trimester of pregnancy, smoking habits during pregnancy, type of occupational activity, educational level, previous parity and miscarriage, and previous episodes of newborn’s low birthweight or preterm delivery). In turn, regression analyses of maternal outcomes at follow-up were adjusted for all the aforementioned variables, as well as for the level of physical exercise (“active” or “inactive” following the same criteria as prior to pregnancy) and the number of new pregnancies during the follow-up; moreover, analyses of child’s post-natal outcomes were adjusted for the child’s level of physical exercise (< or ≥300 min/week) and the type of feeding (breastfeeding, formula or mixed, and length of breastfeeding if applicable). On the other hand, a one-way ANOVA with Bonferroni post hoc analysis was performed to determine differences between groups and sub-groups for continuous data. No intention-to-treat analyses were performed. All statistical analyses were performed using SPSS, version 23.0 software (with the level of significance set at 0.05).

## 3. Results

**Figure 1:**
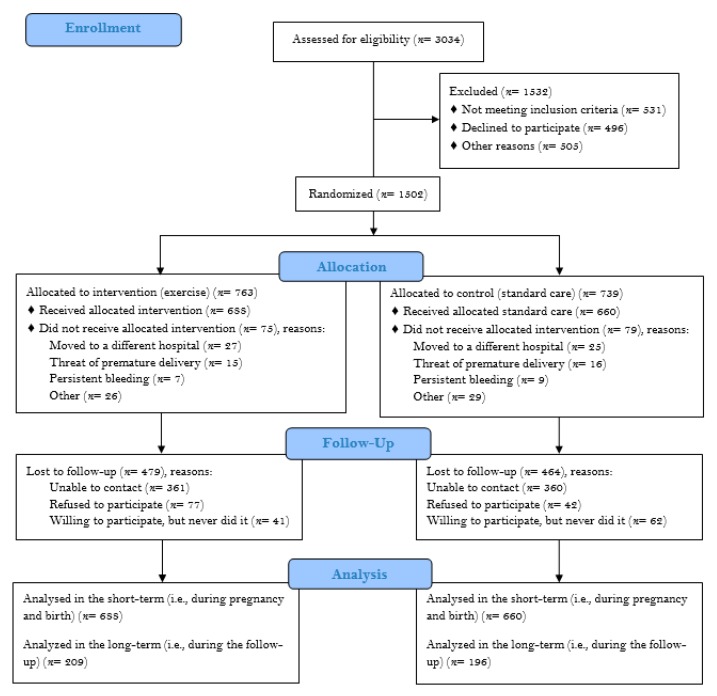
Flowchart of study participants.

We studied 1348 women (all of the same Caucasian (Spanish) descent, 688 in the RCT-pooled intervention (exercise) group (571/117 previously inactive, intervention/previously active, intervention) and 660 controls (98/562 previously active, no intervention/previously inactive, no intervention)) (see participant flow diagram in Figure 1). Groups (Table 1) and subgroups (Table S2, Supplementary Materials) did not essentially differ at baseline. Furthermore, no between-group differences (*p* = 0.132) were found for the levels of previous physical activity (with 15% and 17% of the participants in the control and intervention groups, respectively, considered to be “previously active”).

### 3.1. Adherence and Safety

Adherence to exercise sessions was > 95%. The intervention proved safe for the newborn. Indeed, except for a lower (albeit non-clinically relevant) value in the Apgar score at 5 min in the exercise vs. control group (9.8 ± 0.5 vs. 9.9 ± 0.7, respectively, *p* = 0.038), no differences were found between groups (Table 2) or subgroups (Table S3, Supplementary Materials) in delivery outcomes. Furthermore, the exercise intervention resulted in a lower duration of the first stage of labor compared with the control group (*p* = 0.039) (Table 2).

### 3.2. Maternal and Newborn Outcomes up to Delivery

Compared with controls, exercise during pregnancy significantly reduced the risk of EGWG (adjusted *p* = 0.001), and of both gestational diabetes and hypertension (adjusted *p* = 0.015 and < 0.001, respectively) (Table 3). Sub-group analyses showed the protective effect on gestational diabetes and hypertension (adjusted *p* = 0.042 and 0.001, respectively) to be significant in previously active women who performed the exercise intervention during gestation, but not in those who were previously active and also performed the intervention. In turn, not performing the exercise intervention during pregnancy if being previously active substantially increased (by approximately threefold) the risk for developing gestational hypertension (adjusted *p* = 0.001). Analysis of continuous data showed significantly lower values of SBP (*p* = 0.010), glycemia, and maternal weight gain (both *p* < 0.001) in the exercise group vs. their controls (Table S4, Supplementary Materials).

In the newborn, exercise (vs. the control group) was associated with a lower risk of macrosomia (adjusted *p* = 0.007) and, in subgroup analyses, this protective association remained significant (vs. the reference (“previously inactive, no intervention”) group) only for those women who were previously inactive and performed the exercise intervention during gestation (adjusted *p* = 0.005) (Table 3). No significant differences were found for mean values of newborn’s weight among activity groups or subgroups (all *p* > 0.1, Table S4, Supplementary Materials).

### 3.3. Maternal and Childhood Outcomes at Post-Natal Follow-up

We found a higher likelihood of returning to maternal pre-pregnancy weight within six months postpartum in the exercise vs. the control group (adjusted *p* = 0.007, Table 4). In subgroup analyses, this beneficial association (vs. the reference (“previously inactive, no intervention”) group) remained significant only for the group who was previously inactive and performed the exercise intervention during gestation (adjusted *p* = 0.009).

At the end of follow-up (median 6.1 years (interquartile range 1.8)), exercise during pregnancy (vs. inactive controls) was associated with a lower risk of maternal cardiometabolic conditions other than overweight/obesity or hypertension (adjusted *p* = 0.041) (Table 4, see also Text S1, Supplementary Materials). In subgroup analyses, the association remained significant only for those women who were previously inactive and performed the exercise intervention during pregnancy (adjusted *p* = 0.020). No significant differences were found between groups or subgroup for mean values of maternal SBP, DBP, or BMI (all *p* > 0.1, Table S4, Supplementary Materials).

On the other hand, maternal exercise (vs. control group) was associated with a lower risk of childhood overweight/obesity at one year after birth (adjusted *p* = 0.007) (Table 4, see also Supplementary Table S5, Supplementary Materials). In subgroup analyses, this association only remained significant in the group of women who were previously inactive and performed the exercise intervention during gestation (adjusted *p* = 0.001). Finally, analysis of continuous data showed significantly lower values of BMI and BMI percentile at one year in the children of the exercise vs. control group (Table S5, Supplementary Materials).

## 4. Discussion

The main finding of our study was that supervised, light-moderate gestational exercise following recommendations [14,15] is safe and has benefits for both the mother and the offspring, mainly including protection from the risk of not returning to pre-gestational weight, incident maternal cardiometabolic conditions during and after pregnancy, and newborn’s excess weight up until one year after birth. Another relevant finding was that, at least in women with uncomplicated gestations, pregnancy might represent an optimal time to perform moderate-intensity exercise which, in addition to its safety, might protect their own future health and that of their children. The latter consideration must be emphasized because obstetricians are not always proactive regarding gestational exercise, and they are traditionally concerned about the possibility of potential associated risks [14]. Moreover, sedentary women are usually unlikely to change their exercise habits during pregnancy, and most active women reduce their physical activity levels compared with pre-pregnancy [28].

Our results are in agreement with previous evidence for a potential protective effect of gestational exercise on the risk of EGWG [12,13], gestational hypertension [29], or macrosomia [12], with benefits also reported in overweight/obese women (i.e., lower risk of gestational diabetes or of having large-for-gestational-age newborns) [30,31]. In this respect, a novel finding of our study was that the benefits of gestational exercise on maternal and child health were overall maximized in those who were previously inactive, as they had approximately one-third the risk of gestational hypertension than their previously inactive peers who did not perform the exercise intervention. Of note, the lack of significant benefits observed for those women who were previously active and participated in the intervention compared to their previously inactive peers performing the same intervention might be potentially explained by to the lower sample size of the former group. In turn, maternal exercise seems to benefit the maintenance of a healthy weight after pregnancy in both the mother and child, with this effect particularly strong in those previously inactive women who exercised during gestation. Finally, maternal exercise was associated with a lower risk of incident maternal cardiometabolic conditions other than hypertension or obesity at approximately six years follow-up, and the benefit was very strong in those who were previously inactive but exercised during pregnancy (~80% lower risk). In this regard, scarce data are available in the literature on the long-term effects of maternal exercise in maternal or childhood health [32,33]. On the other hand, more research is needed to unveil the biological mechanisms that are responsible for the protective effects of gestational exercise against macrosomia or overweight/obesity at one year in the offspring. Potential factors are reductions in insulin-like growth factor 1 and in endocrine stimulation to fetal growth in general, together with modulation of maternal insulin sensitivity [32].

In addition to the numerous losses at follow-up, the main limitations of our study were lack of control for maternal/child dietary habits and the fact that women’s exercise habits were self-reported for pre- and post-pregnancy, and not assessed during pregnancy (apart from the exercise intervention). Moreover, reasons for being—or not—active during pre-pregnancy could have been related to health conditions (e.g., overweight). On the other hand, postpartum maternal weight was also self-reported. However, weight data were found to accurately represent actual values abstracted from medical records in reproductive-aged women [34]. In turn, a main strength was a previously unexplored assessment of both pre-pregnancy and pregnancy activity habits on important maternal and child health outcomes up to delivery and at post-natal follow-up. Furthermore, the exercise intervention was professionally supervised, resulting in a very high adherence. Finally, follow-up assessment was adjusted for relevant potential confounders (including maternal/child postpartum exercise habits, breastfeeding, or subsequent pregnancies).

## 5. Conclusions

In summary, the gestational exercise intervention we used proved safe and beneficial for both the mother and the child, allowing them to maintain an overall healthier cardiometabolic status. Our findings are relevant in the light of the growing incidence of EGWG, which is contributing to the intergenerational transmission of obesity [35], and they reinforce the notion that pregnancy should no longer be considered a state of confinement. In fact, it might represent an optimal time to exercise at moderate intensities.

## Figures and Tables

**Table 1:** Participants’ baseline characteristics in the intervention (exercise) and control groups.

	Control (*n* = 660)	Exercise (*n* = 688)	*p*-Value
Age (years)	31 ± 4	32 ± 4	0.168
SBP (mmHg)	110 ± 12	108 ± 9	0.077
DBP (mmHg)	64 ± 8	63 ± 7	0.584
Pre-gestational weight (kg)	63 ± 12	64 ± 11	0.103
Pre-gestational BMI (kg·m^−2^)	23.6 ± 4.0	23.5 ± 3.9	0.534
Pre-gestational BMI category			0.349
Underweight (<18.5 kg·m^−2^)	5%	4%	
Normal weight (18.5–24.9 kg·m^−2^)	67%	69%	
Overweight (25–29.9 kg·m^−2^)	20%	21%	
Obese (>30 kg·m^−2^)	7%	7%	
Smoking during pregnancy	17%	15%	0.353
Occupational activity			**<0.001**
Housewife	19%	27%	
Sedentary job	55%	35%	
Active job	26%	38%	
Educational level			0.074
Primary	26%	23%	
Higher	43%	40%	
University	31%	37%	
Parity			0.703
Nulliparous	58%	59%	
1 before	36%	34%	
2+ before	6%	7%	
Previous miscarriage			0.224
No	73%	73%	
1 before	23%	21%	
2+ before	4%	6%	
Previous low birthweight	2%	2%	0.361
Previous preterm delivery	4%	3%	0.451

Data are shown as means ± SD or frequency (%). Abbreviation: BMI, body mass index; DBP, diastolic blood pressure; SBP, systolic blood pressure. Significant *p*-values (< 0.05) are in bold. SBP and DBP values correspond to the first prenatal visit.

**Table 2:** Delivery endpoints by group.

	Control (*n* = 660)	Exercise (*n* = 688)	*p*-value
Gestational age (days)	277 ± 10	277 ± 12	0.776
Preterm delivery	5%	5%	0.671
Birthweight (g)	3265 ± 459	3234 ± 457	0.232
Apgar score 1 min	8.7 ± 1.2	8.8 ± 1.2	0.701
Apgar score 5 min	9.9 ± 0.7	9.8 ± 0.5	**0.038**
Instrumental delivery	16%	15%	0.482
Cesarean delivery	22%	19%	0.288
Duration stage 1 of labor (min)	430 ± 501	382 ± 256	**0.039**
Duration stage 2 of labor (min)	45 ± 50	49 ± 53	0.199
Duration stage 3 of labor (min)	8 ± 7	9 ± 11	0.060

Data are means ± SD or %. Significant *p*-values (<0.05) are in bold.

**Table 3:** Association of pre-pregnancy and pregnancy exercise with study outcomes up to delivery.

Outcome	Overall Analysis ^a^			Sub-Group Analysis ^a^						
	Control (*n* = 660) [Reference]	Exercise (*n* = 688)		Previously Inactive, no Intervention (*n* = 562) Reference	Previously Active, Intervention (*n* = 117)		Previously Active, no Intervention (*n* = 98)		Previously Inactive, Intervention (*n* = 571)	
		Crude OR (95% CI)	Adjusted OR (95% CI) ^b^		Crude OR (95% CI)	Adjusted OR (95% CI) ^b^	Crude OR (95% CI)	Adjusted OR (95% CI) ^b^	Crude OR (95% CI)	Adjusted OR (95% CI) ^b^
Mother										
Gestational hypertension	[1]	**0.43** **(0.26–0.71)** ***p* = 0.001**	**0.39** **(0.23–0.67)** ***p* < 0.001**	[1]	1.40 (0.67–2.92) *p* = 0.364	1.37 (0.63–2.98) *p* = 0.358	**2.91** **(1.54–5.50)** ***p* = 0.001**	**3.00** **(1.54–5.83)** ***p* = 0.001**	**0.38** **(0.20–0.71)** ***p* = 0.003**	**0.33** **(0.17–0.65)** ***p* = 0.001**
Gestational diabetes	[1]	**0.53** **(0.31–0.89)** ***p* = 0.016**	**0.48** **(0.28–0.84)** ***p* = 0.015**	[1]	0.42 (0.13–1.40) *p* = 0.157	0.35 (0.10–1.21) *p* = 0.174	1.21 (0.52–2.82) *p* = 0.657	1.06 (0.44–2.55) *p* = 0.723	0.57 (0.32–1.00) *p* = 0.057	**0.52** **(0.28–0.95)** ***p* = 0.042**
EGWG	[1]	**0.66** **(0.52–0.84)** ***p* = 0.001**	**0.60** **(0.46–0.79)** ***p* = 0.001**	[1]	**0.51** **(0.31–0.83)** ***p* = 0.007**	**0.50** **(0.29–0.87)** ***p* = 0.011**	1.24 (0.80–1.95) *p* = 0.340	1.20 (0.73–1.99) *p* = 0.403	**0.72** **(0.56–0.94)** ***p* = 0.014**	**0.64** **(0.48–0.86)** ***p* = 0.007**
Newborn										
Low birth weight (<2500 g)	[1]	0.79 (0.48–1.30) *p* = 0.413	0.80 (0.46–1.37) *p* = 0.474	[1]	0.59 (0.21–1.71) *p* = 0.362	0.58 (0.19–1.73) *p* = 0.204	0.52 (0.16–1.74) *p* = 0.316	0.54 (0.16–1.85) *p* = 0.226	0.76 (0.44–1.30) *p* = 0.371	0.78 (0.44–1.39) *p* = 0.427
Macrosomia (>4000 g)	[1]	**0.43** **(0.25–0.73)** ***p* = 0.002**	**0.36** **(0.20–0.63)** ***p* = 0.007**	[1]	0.48 (0.17–1.37) *p* = 0.168	0.47 (0.16–1.39) *p* = 0.204	0.88 (0.36–2.13) *p* = 0.764	0.89 (0.35–2.22) *p* = 0.326	**0.41** **(0.23–0.74)** ***p* = 0.003**	**0.33** **(0.18–0.62)** ***p* = 0.005**

Data are odds ratios (ORs) and 95% confidence intervals (CIs). Significant associations (*p* < 0.05) are in bold. Participants were placed in the four subgroups based on their previous physical activity habits and group assignment in RCT during pregnancy (intervention (exercise)/control), with women considered to be “previously active” if they reported in the first prenatal visit having exercised on three or more days per week during the previous year, or to be “previously inactive” otherwise [19]. Abbreviations: EGWG, excessive gestational weight gain (i.e., >18 kg for underweight women, >16 kg for normal weight, >11.5 kg for overweight, and >9 kg for obese) [21]). Symbols: ^a^ the overall analysis refers to randomized allocation during pregnancy (intervention (exercise) vs. control group, respectively) whereas sub-group analyses also consider exercise habits in the year before pregnancy; ^b^ adjusted for all the variables shown in Table 1 (maternal age, pre-pregnancy BMI category, gestational hypertension during the first trimester of pregnancy, smoking habits during pregnancy, type of occupational activity, educational level, previous parity and miscarriage, and previous episodes of newborn’s low birthweight or preterm delivery).

**Table 4:** Association of pre-pregnancy and pregnancy exercise with study outcomes at post-natal follow-up.

Outcome	Overall Analysis ^a^			Sub-Group Analysis ^a^								
	Control (*n* = 196) Reference	Exercise (*n* = 209)		Previously Inactive, no Intervention (*n* = 165) Reference	Previously Active, Intervention (*n* = 40)		Previously Active, no Intervention (*n* = 31)		Previously Inactive, Intervention (*n* = 169)			
		Crude OR (95% CI)	Adjusted OR (95% CI) ^b^		Crude OR (95% CI)	Adjusted OR (95%CI) ^b^	Crude OR (95% CI)	Adjusted OR (95% CI) ^b^	Crude OR (95% CI)	Adjusted OR (95% CI) ^b^		
Mother												
Return to pre-pregnancy weight within 6 months	[1]	1.58 (0.98–2.55) *p = 0.059*	**2.37** **(1.26–4.54)** ***p* = 0.007**	[1]	1.07 (0.48–2.38) *p = 0.875*	1.37 (0.45–4.11) *p = 0.580*	0.89 (0.32–2.48) *p = 0.822*	0.63 (0.17–2.32) *p = 0.483*	**1.71** **(1.01–2.89)** ***p* = 0.045**	**2.44** **(1.25–4.75)** ***p* = 0.009**		
Overweight/obesity	[1]	0.80 (0.53–1.22) *p = 0.307*	0.57 (0.28–1.19) *p = 0.136*	[1]	0.48 (0.21–0.10) *p = 0.083*	0.88 (0.21–3.73) *p = 0.862*	0.92 (0.40–2.10) *p = 0.845*	1.83 (0.54–6.17) *p = 0.332*	0.88 (0.56–1.39) *p = 0.585*	0.67 (0.21–1.35) *p = 0.219*		
Hypertension (≥140/90 mmHg)	[1]	0.44 (0.13–1.51) *p = 0.192*	0.97 (0.12–7.74) *p = 0.976*	[1]	−	−	0.88 (0.10–7.59) *p = 0.904*	4.59 (0.20–108.13) *p = 0.345*	0.54 (0.15–1.90) *p = 0.335*	1.57 (0.15–16.17) *p = 0.704*		
Hypertension (≥130/80 mmHg)	[1]	0.58 (0.32, 1.07) *p* = 0.083	0.75 (0.35, 1.60) *p* = 0.454	[1]	0.25 (0.06, 1.14) *p* = 0.073	0.30 (0.05, 1.72) *p* = 0.177	0.89 (0.27, 2.96) *p* = 0.848	0.67 (0.15, 2.95) *p* = 0.601	0.66 (0.34, 1.26) *p* = 0.207	0.78 (0.35, 1.76) *p* = 0.551		
Cardiometabolic conditions	[1]	**0.19** **(0.06–0.58)** ***p* = 0.003**	**0.27** **(0.08–0.95)** ***p* = 0.041**	[1]	0.24 (0.03–1.89) *p = 0.177*	0.64 (0.06–6.47) *p = 0.708*	0.64 (0.14–.93) *p = 0.563*	0.29 (0.03–2.70) *p = 0.280*	**0.17** **(0.05–0.59)** ***p* = 0.005**	**0.19** **(0.05–0.76)** ***p* = 0.020**		
Child												
Overweight/obesity at 1 year	[1]	**0.31** **(0.15–0.62)** ***p* = 0.001**	**0.20** **(0.06–0.63)** ***p* = 0.007**	[1]	0.34 (0.10–1.22) *p = 0.099*	0.37 (0.06–2.13) *p = 0.265*	0.30 (0.07–1.37) *p = 0.121*	−	**0.25** **(0.12–0.55)** ***p* < 0.001**	**0.06** **(0.01–0.30)** ***p* = 0.001**		
Overweight/obesity at the end of follow-up	[1]	0.89 (0.50–1.59) *p = 0.702*	0.57 (0.23–1.38) *p = 0.210*	[1]	0.61 (0.20–1.90) *p = 0.396*	0.39 (0.08–1.85) *p = 0.235*	0.56 (0.16–1.99) *p = 0.366*	0.97 (0.17–5.54) *p = 0.969*	0.87 (0.47–1.62) *p = 0.667*	0.61 (0.23–1.59) *p = 0.310*		
Low weight at 1 year	[1]	2.68 (0.28–26.04) *p = 0.397*	−	[1]	−	−	−	−	−	−		
Low weight at the end of follow-up	[1]	1.16 (0.54–2.50) *p = 0.702*	0.41 (0.12–1.44) *p = 0.166*	[1]	0.39 (0.05–3.19) *p = 0.383*	0.22 (0.02–2.63) *p = 0.234*	1.58 (0.40–6.15) *p = 0.513*	1.00 (0.08–12.28) *p = 1.000*	1.49 (0.64–3.44) *p = 0.352*	0.47 (0.12–1.79) *p = 0.268*		
Cardiometabolic conditions at the end of follow-up	[1]	1.11 (0.37–3.36) *p = 0.856*	0.55 (0.11–2.68) *p = 0.455*	[1]	0.86 (0.10–7.58) *p = 0.892*	1.45 (0.09–23.06) *p = 0.792*	1.06 (0.12–9.40) *p = 0.958*	−	1.18 (0.35-3.94) *p = 0.790*	0.39 (0.07–2.23) *p = 0.287*		

Data are odds ratios (ORs) and 95% confidence intervals (CIs). Significant associations (*p* < 0.05) are in bold. Participants were placed in the four subgroups based on their previous exercise habits and group assignment in randomized controlled trial (RCT) during pregnancy (intervention (exercise)/control), with women considered to be “previously active” if they reported in the first prenatal visit having exercised on three or more days per week during the previous year, or to be “previously inactive” otherwise [19]. Except for return to pre-pregnancy weight within six months, all maternal outcomes correspond to the end of the follow-up. Symbols: − the analysis could not be performed due to insufficient number of cases; ^a^ the overall analysis refers to randomized allocation during pregnancy (intervention (exercise) vs. control group, respectively), whereas sub-group analyses also consider exercise habits in the year before pregnancy; ^b^ adjustment variables were maternal age, pre-pregnancy body mass index category, gestational hypertension during the first trimester of pregnancy, smoking habits during pregnancy, type of occupational activity, educational level, previous parity and miscarriage, and previous episodes of newborn’s low birthweight or preterm delivery, level of physical exercise and the number of new pregnancies during the follow-up, as well as child’s level of physical exercise and the type of feeding for the analyses of child’s post-natal outcomes.

## Supplementary Materials

The following are available online at https://www.mdpi.com/2077-0383/9/2/379/s1: Text S1: Types of incident cardiometabolic conditions recorded at the end of follow-up; Figure S1: Examples of exercise; Video S1: Examples of exercises; Table S1: Maternal exercise training distribution in each trimester of pregnancy; Table S2: Participants’ baseline characteristics by sub-group; Table S3: Newborn and delivery endpoints by subgroup; Table S4: Continuous data up to delivery by group and subgroup; Table S5: Continuous data at post-natal follow-up by group and subgroup.
