# Supplementary material for: Gestational Exercise and Maternal and Child Health: Effects until Delivery and at Post-Natal Follow-up
Source: J Clin Med. 2020 Jan 31;9(2):379. doi: 10.3390/jcm9020379 (PMC7074577; doi:10.3390/jcm9020379)
Supplement: Supplementary file 1 [file jcm-09-00379-s001.zip › jcm-684070-SI.docx]

**Supplementary text 1.** Types of incident cardiometabolic conditions recorded at the end of follow-up

The following conditions other than hypertension or obesity/overweight were recorded: in the mothers (*n* = 22 cases in total), thyroid disease and cardiovascular disease (thromboembolism, venous insufficiency, thrombosis); in the children (n= 16 cases): thyroid disease, heart murmur, and congenital heart disease. No case of postnatal incident diabetes was found among mothers or children.

**Supplementary Figure 1.**

Resistance training exercises


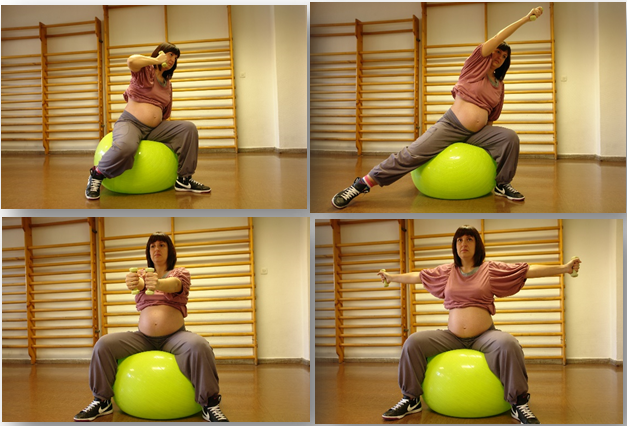


Stretching exercises


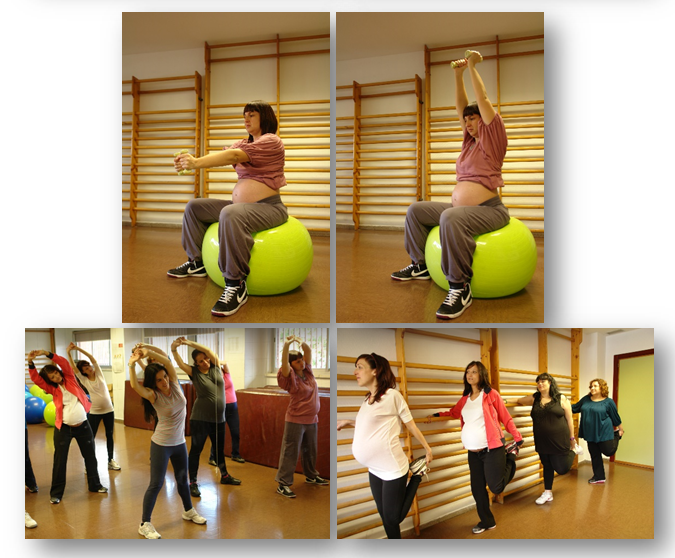


**Supplementary Table 1**. Maternal exercise training distribution in each trimester of pregnancy.

Each session included an initial warm-up and a final cool down period (≈ 5 min each).

|  | **Late 1st trimester** | **2nd trimester** | **3rd trimester** |
| --- | --- | --- | --- |
| **Low-impact dance**:  Intensity equivalent to less than 60% of age-predicted maximum heart rate (220 minus age, in years) and individual rating of perceived exertion ranging from 10 to 12 (corresponding to ‘fairly light’ to ‘somewhat hard’, respectively) | Achieved aerobic intensity | Maintained aerobic intensity | Maintained aerobic intensity |
| **Resistance exercises**:  Barbell exercises (up to 3 kg).  Low to medium resistance band exercises | Barbell exercises (1-2 kg).  Low resistance band exercises | Barbell exercises (2-3 kg).  Medium resistance band exercises | Barbell exercises  (2-3 kg).  Medium resistance band exercises |
| **Stretching exercises**:  Including large upper and lower-body muscles | Maintained stretching load | Maintained stretching load | Maintained stretching load |

**Supplementary Table 2.** Participants’ baseline characteristics by sub-group.

|  | **‘Became active’**  **(n=571)** | **‘Remained active’**  **(n=117)** | **‘Remained inactive’**  **(n=562)** | **‘Became inactive’**  **(n=98)** | **p-value for subgroup effect** |
| --- | --- | --- | --- | --- | --- |
| **Age (years)** | 32 ± 4 | 32 ± 4 | 31 ± 4 | 32 ± 5 | .487 |
| **Pre-gestational weight (kg)** | 63.6 ± 11.2 | 63.3 ± 10.5 | 62.3 ± 11.3 | 64.0 ± 13.2 | .208 |
| **Pre-gestational BMI (kg·m^-2^)** | 23.5 ± 4.0 | 23.2 ± 3.8 | 23.5 ± 4.0 | 24.1 ± 4.3 | .474 |
| **Pre-gestational BMI category n (%)** |  |  |  |  | .589 |
| Underweight (<18.5 kg·m^-2^) | 4% | 3% | 5% | 6% |  |
| Normal weight (18.5–24.9 g·m^-2^) | 68% | 75% | 68% | 62% |  |
| Overweight (25–29.9 kg·m^-2^) | 22% | 15% | 19% | 23% |  |
| Obese (>30 kg·m^-2^) | 7% | 6% | 7% | 8% |  |
| **Smoking during pregnancy (%)** | 15% | 14% | 17% | 13% | .595 |
| **Occupational activity (%)** |  |  |  |  | **< .001** |
| Housewife | 25% | 33% | 19% | 21% |  |
| Sedentary job | 36% | 32% | 51% | 53% |  |
| Active job | 39% | 35% | 26% | 27% |  |
| **Educational level (%)** |  |  |  |  | .172 |
| Primary | 23% | 26% | 25% | 29% |  |
| Higher | 38% | 43% | 43% | 43% |  |
| University | 39% | 31% | 32% | 29% |  |
| **Parity n (%)** |  |  |  |  | .753 |
| Nulliparous | 59% | 62% | 58% | 60% |  |
| One | 34% | 32% | 35% | 37% |  |
| Two or more | 7% | 6% | 7% | 3% |  |
| **Previous miscarriage (%)** |  |  |  |  | .635 |
| No | 74% | 69% | 73% | 72% |  |
| One | 20% | 24% | 23% | 23% |  |
| Two or more | 5% | 7% | 4% | 4% |  |
| **Previous low birthweight (%)** | 2% | - | 2% | 1% | .137 |
| **Previous preterm delivery (%)** | 3% | 3% | 4% | 3% | .903 |

Data are mean ± SD or %. Abbreviation: BMI, body mass index. Significant p-value (< .05) is in bold.

**Supplementary Table 3.** Newborn and delivery endpoints by subgroup.

|  | **Remained inactive**  **(n=562)** | **Remained active**  **(n=117)** | **Became inactive**  **(n=98)** | **Became active**  **(n=571)** | **p-value for subgroup effect** |
| --- | --- | --- | --- | --- | --- |
| Gestational age (days) | 277 ± 10 | 278 ±10 | 277 ± 10 | 277 ± 12 | .958 |
| Preterm delivery (%) | 5% | 8% | 6% | 4% | .370 |
| Birthweight (g) | 3,258 ± 461 | 3,282 ± 436 | 3,275 ± 445 | 3,227 ± 462 | .485 |
| Apgar score 1 min | 8.8 ± 1.2 | 8.7 ± 1.5 | 8.7 ± 1.2 | 8.8 ± 1.2 | .844 |
| Apgar score 5 min | 9.9 ± 0.6 | 9.8 ± 0.6 | 9.9 ± 1.1 | 9.8 ± 0.5 | .175 |
| Instrumental delivery (%) | 17% | 16% | 14% | 15% | .770 |
| Cesarean delivery n (%) | 22% | 13% | 20% | 21% | .170 |
| Duration stage 1 of labor (min) | 430 ± 526 | 366 ± 233 | 431 ± 324 | 385 ± 260 | .217 |
| Duration stage 2 of labor (min) | 47 ± 53 | 45 ± 46 | 34 ± 29 | 50 ± 55 | .105 |
| Duration stage 3 of labor (min) | 8 ± 6 | 7 ± 6 | 8 ± 11 | 10 ± 12 | .070 |

Data are mean ± SD or frequency (%).

**Supplementary Table 4.** Continuous data up to delivery by group and subgroup.

| **Outcome** | **Overall analysis** | | | **Sub-group analysis** | | | | |
| --- | --- | --- | --- | --- | --- | --- | --- | --- |
|  | **Control**  **(n=660)** | **Exercise**  **(n=688)** | **p-value** | **Remained inactive**  **(n=562)** | **Remained active**  **(n=117)** | **Became inactive**  **(n=98)** | **Became active**  **(n=571)** | **p-value for subgroup effect** |
| **Mother** | | | | | | | | |
| SBP  (mmHg) | 116 ± 12 | 114 ± 11 | **.010** | 115± 12* | 116 ± 12 | 119 ± 14 | 114 ± 11† | **< .001** |
| DBP (mmHg) | 69 ± 10 | 68 ± 9 | .127 | 69 ± 10 | 68 ± 10 | 71 ± 11 | 68 ± 9 | .056 |
| Glycaemia  (mg/dL) | 122 ± 30 | 115 ± 28 | **< .001** | 123 ± 30 | 111 ± 27‡ | 118 ± 32 | 116 ± 28^#^ | **< .001** |
| Weight gain (kg) | 13.3 ±4.3 | 12.3 ± 3.8 | **< .001** | 13.3 ± 4.3 | 12.3 ± 3.8 | 13.5 ± 4.0 | 12.3 ± 3.9 † | **< .001** |
| **Newborn** | | | | | | | | |
| Body weight (g) | 3,273 ± 458 | 3,239 ± 452 | .158 | 3,269 ± 459 | 3,269 ± 415 | 3,297 ± 453 | 3,233 ± 460 | .383 |

Data are mean ± SD. Significant p values (< 0.05) are in bold. Abbreviations: DBP, diastolic blood pressure; SBP, systolic blood pressure. *p = .015 *vs* ‘became inactive’; †p < .001 *vs* ‘became inactive’; ‡ p < .001 *vs* ‘remained inactive’.

**Supplementary Table 5.** Continuous data at postnatal follow-up by group and subgroup.

| **Outcome** | **Overall analysis** | | | **Sub-group analysis** | | | | |
| --- | --- | --- | --- | --- | --- | --- | --- | --- |
|  | **Control**  **(n = 196)** | **Exercise**  **(n = 209)** | **p-value** | **Remained inactive**  **(n= 165)** | **Remained active**  **(n = 40)** | **Became inactive**  **(n = 31)** | **Became active**  **(n = 169)** | **p-value** |
| **Mother** | | | | | | | | |
| SBP(mmHg) | 112 ± 15 | 110 ± 11 | .333 | 112 ± 14 | 108 ± 11 | 111 ± 20 | 111 ± 11 | .510 |
| DBP (mmHg) | 69 ± 10 | 68 ± 9 | .382 | 69 ± 10 | 65 ± 9 | 68 ± 9 | 69 ± 10 | .326 |
| BMI (kg·m^2^) | 24.5 ± 5.3 | 23.9 ± 4.4 | .192 | 24.5 ± 4.9 | 23.1 ± 3.3 | 24.7 ± 6.9 | 24.0 ± 4.6 | .372 |
| **Child** | | | | | | | | |
| BMI at 1 year (kg·m^-2^) | 17.0 ± 1.3 | 16.7 ± 1.2 | **.022** | 17.1 ± 1.3* | 16.7 ± 1.2 | 16.6 ± 1.4 | 16.6 ± 1.2 | **.037** |
|  |  |  |  |  |  |  |  |  |
|  |  |  |  |  |  |  |  |  |
| BMI percentile at end of follow-up (%) | 50th | 47th | .269 | 51th | 45th | 45th | 47th | .525 |

Data are mean ± SD. Significant p-values (< .05) are in bold. Abbreviations: BMI, body mass index; DBP, diastolic blood pressure; SBP, systolic blood pressure. *p = .043 *vs* ‘became active’. All maternal outcomes correspond to end of follow-up.
